# Invasion and Persistence of a Selfish Gene in the Cnidaria

**DOI:** 10.1371/journal.pone.0000003

**Published:** 2006-12-20

**Authors:** Matthew R. Goddard, Jessica Leigh, Andrew J Roger, Andrew J Pemberton

**Affiliations:** 1 School of Biological Sciences, University of Auckland New Zealand; 2 National Environment Research Council Centre for Population Biology, Imperial College London, United Kingdom; 3 Department of Biochemistry and Molecular Biology, Dalhousie University Halifax, Nova Scotia, Canada; 4 Marine Biological Association of the United Kingdom Plymouth, United Kingdom; University of Queensland, Australia

## Abstract

**Background:**

Homing endonuclease genes (HEGs) are superfluous, but are capable of invading populations that mix alleles by biasing their inheritance patterns through gene conversion. One model suggests that their long-term persistence is achieved through recurrent invasion. This circumvents evolutionary degeneration, but requires reasonable rates of transfer between species to maintain purifying selection. Although HEGs are found in a variety of microbes, we found the previous discovery of this type of selfish genetic element in the mitochondria of a sea anemone surprising.

**Methods/Principal Findings:**

We surveyed 29 species of Cnidaria for the presence of the *COXI* HEG. Statistical analyses provided evidence for HEG invasion. We also found that 96 individuals of *Metridium senile*, from five different locations in the UK, had identical HEG sequences. This lack of sequence divergence illustrates the stable nature of Anthozoan mitochondria. Our data suggests this HEG conforms to the recurrent invasion model of evolution.

**Conclusions:**

Ordinarily such low rates of HEG transfer would likely be insufficient to enable major invasion. However, the slow rate of Anthozoan mitochondrial change lengthens greatly the time to HEG degeneration: this significantly extends the periodicity of the HEG life-cycle. We suggest that a combination of very low substitution rates and rare transfers facilitated metazoan HEG invasion.

## Introduction

Homing endonuclease genes (HEGs) are superfluous, but invade populations because they cheat the rules of Mendelian inheritance and over-represent themselves in the next generation [1]–[3]. Each homing endonuclease (HE), the protein product of an HEG, recognises and cuts a specific 15–30 bp DNA recognition sequence which is typically unique within a genome [4], [5]. HEGs are located in the middle of their own recognition sequence and so their presence splits this site in two and protects against chromosome cleavage. However, in individuals heterozygous for any particular HEG, the uninterrupted recognition sequence is encountered and cut producing a potentially lethal broken chromosome [4]. Chromosome breaks are typically repaired using the homologous area of the intact chromosome as a template, and the HEG is copied onto the other chromosome as a consequence of the repair procedure [1]. This ‘homing’ process allows HEGs to effect super-Mendelian inheritance and increase in frequency [2].

The infective nature of HEGs allows them to quickly invade species [2], but the lack of homing opportunities near and at fixation means that selection for HEG function will become substantially relaxed. One evolutionary model suggests that purifying selection may only be maintained, and thus degeneration circumvented, by restoring homing events [6]. The recurrent invasion of closely related uninfected species, via horizontal transfer, is a hypothesis that permits the longer-term persistence of HEGs by maintaining purifying selection. This cyclical evolutionary model allows major invasions of closely related species, and predicts the patchy distribution of both functional and non-functional HEGs, and incongruence between host-genome and HEG phylogenies [6]–[8]. HEGs have been reported from a wide array of species and comparative data from phage, archaea, fungi, algae and plants support this HEG recurrent invasion model (see [1] and references within). One clear prediction of this model is that selection will produce HEGs that are adapted for major invasion by having recognition sites that are conserved among taxa. This seems to be the case since extant HEGs have recognition sequences usually targeted toward essential and sequentially conserved genes [7]. The presence of HEGs in the nuclear genomes of eukaryotes, archebacteria and phage correlates with the fact that alleles may be bought together in each of these groups by mating, conjugation and co-infection respectively, and thus permit HEG homing and invasion [1], [3], [5], [9], [10]. If HEG-plus and HEG-minus alleles are not bought together there will be no opportunities for homing and spread within a species. In general HEGs will not be able to persist in strictly asexual genomes [2]. Thus, it appears that HEGs have two requirements for major invasion: 1, a reasonable opportunity for homing; and 2, relatively frequent transfer to naive hosts to maintain purifying selection and escape degeneration.

An HEG has been discovered in the cytochrome oxidase I (*COXI*) gene of the mitochondria of the sea anemone *Metridium senile* [11]. This HEG is located in the middle of a self splicing group I intron (as is common) which allows excision from the host gene at the RNA level: this allows the production of a functional COXI. This original report of an HEG among metazoans is one of an extremely small number of known metazoan mitochondrial introns. This discovery is puzzling. The mitochondria of animals are generally thought to be uni-parentally inherited, and not to recombine: this should deny opportunities for homing. Even if within-species HEG spread were possible for some reason, then escape from long-term degeneration would seem unlikely as one imagines horizontal transfer between metazoan germ-lines to be a prohibitively rare event.

We investigated the distribution and evolutionary genetics of this HEG within the Cnidaria in order to test the applicability of the recurrent invasion model [6] in metazoans. We present evidence which strongly suggests that this HEG invaded and then spread within the Actinaria (true sea anemones).

## Results

### Phylogenies and HEG distribution

**Figure 1:**
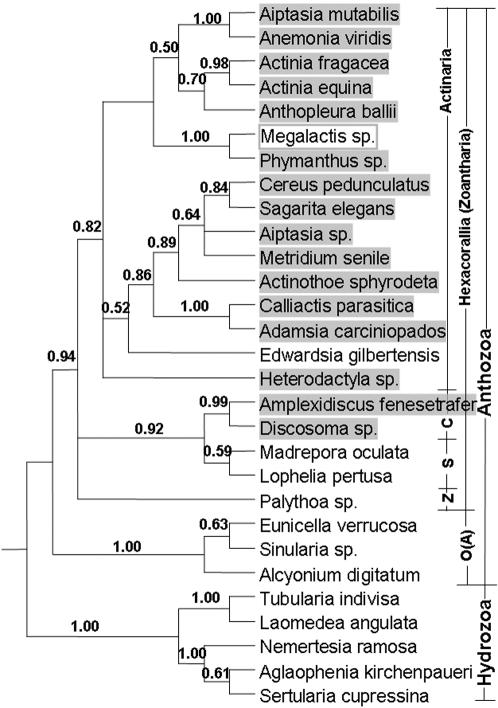
Cnidaria host genome phylogeny reconstructed using Bayesian methods from 18S, 5.8S and ITS data, and is a 50% majority rule consensus taken from the MCMC analyses after plateau (i.e. ‘burn-in’ trees discarded). The Bayesian posterior probabilities are given for each node. Taxa containing a HEG are highlighted; the non-functional HEG is boxed. Taxonomic classification is also distinguished; O(A)  =  Octocorallia (Alcyonaria); C  =  Corrimorpharia; S  =  Scleratinia; Z  =  Zoanthiniaria.

The nuclear 18S, 5.8S and ITS sequence data were assumed to represent the evolutionary history of the Cnidarian host genome (accession numbers 18S: DQ831237 - DQ831296; ITS1: DQ831297 - DQ831306; 5.8S: DQ831307 - DQ831322; ITS2: DQ831323 - DQ831332). After alignment and correct model selection (see Supporting Material 1), Bayesian, likelihood and parsimony optimality criteria produced extremely similar phylogenetic trees; the tree recovered using Bayesian methods is shown in figure 1. The partitioning of the taxa in this phylogeny agrees with previous estimates of Cnidarian phylogeny [12]–[14], and with their taxonomic classifications.

All twenty nine taxa produced a single *COXI* PCR product, and seventeen of these harboured the correct sized larger product indicating the presence of an HEG in the *COXI* gene. When HEG presence is mapped onto the host phylogeny the distribution appears non-random, with a strong cluster in members of the Actinaria and Corrimorpharia (figure 1). Such a pattern seems to minimise the change of HEG status across the tree, and implies that horizontal transfer has not played a large part in this HEG's evolutionary history. Indeed, if we assume that HEG gains and losses are equally likely, then the inferred three character changes across the tree (two gains and one loss) are significantly less than that observed when intron status is randomised (*P*<0.01; *n* = 100 randomisations). This clumped distribution, and simple test, suggests this HEG has not been subject to rampant horizontal transfer across all these taxa, and, for some reason, that the HEG is restricted to specific clades.

### Tests for HEG horizontal transfer

**Figure 2:**
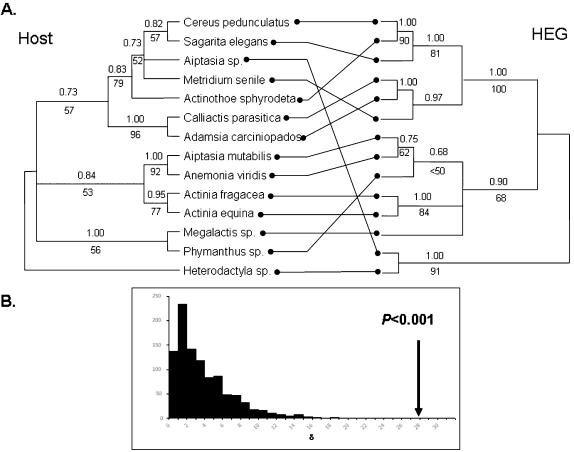
Comparison of Actinarian and HEG phylogenies. **A.** The numbers above each branch are the Bayesian posterior probabilities (again after burn-in trees discarded), and those below the branch are the support generated from a likelihood bootstrap analysis (n = 1000). The HEG tree is unrooted (it is unknown) and displayed in such a way as to minimise the differences with the host tree. **B.** The null distribution for the likelihood ratio test generated by evaluating δ for 1000 non-parametric bootstrap resampled data sets, 50% of which are drawn entirely from the host data and 50% from the HEG data. The likelihood ratio for the observed partition is shown.

A more robust test for horizontal transfer makes use of sequence data from both areas of interest. We obtained sequence for fourteen of the HEGs and their flanking group I introns (accession numbers DQ831333 - DQ831345). All HEGs were putatively functional, with the exception of that found in *Megalactis sp*. which had four deletions of between four and 12 bp which destroyed the HEG reading frame (one deletion even removes the HEG start codon). Overall the HEG sequences showed extremely good alignment allowing un-ambiguous translation for all but the non-functional version in *Megalactis sp*. The protein motifs which place this HEG in the LAGLIDADG family [9] may be readily identified. Phylogenies reconstructed exclusively from the Actinarian derived HEGs using Bayesian, likelihood and parsimony criteria all agreed (see Supporting Material 1 for model details). A phylogeny reconstructed from the translated amino acid data was also congruent with the DNA derived phylogenies. Statistical comparisons of host and HEG data allowed us clear tests for differences in evolutionary histories. An Actinaria host tree was reconstructed using only those taxa for which we have HEG sequence (see figure 2A). First, this was compared to the HEG tree using the likelihood based Shimodaira-Hasegawa (SH) test as implemented by PAUP* (full optimisation, 1000 replicates). Comparisons performed on each relevant data set returned a *P* value of <0.001 in each case (four tests total, bootstrap and best trees tested on both data partitions) which suggested that the two data sets contained different phylogenetic signals. One criticism with the SH tests, as carried out here, is that only two topologies were considered at a time: the test was really designed to consider a large multiplicity of trees [15]. In addition, the support for some of the nodes seen in figure 2 is low – ideally tests which do not rely on fixed topologies should be employed.

A more appropriate test for incongruence uses Bayes factors (BF) to compare evolutionary histories [16]. The marginal log-likelihood of an analysis where the HEG and the host partitions were forced to share the same topology (the ‘one tree’ model) was subtracted from the marginal log-likelihood of an analysis which allowed a separate tree from each partition (the ‘two tree’ model): this yielded an estimate of the log-Bayes factor. Four separate MCMCMC runs of the ‘one’ tree versus ‘two tree’ models were completed, and the resulting average log-BF was 23.48. This test circumvents the problems mentioned above, and provides strong evidence against accepting the simpler one tree model since 2(log-BF) >10 (see [16]): the two tree model is clearly favoured. However, some caution is warranted in interpreting this result, as a recent analysis suggests that the harmonic mean estimate of the marginal log-likelihood for these Bayes factor calculations may be biased in favour of more complex models [17].

Thus, as a final test for incongruence between HEG and host histories, we used a non-parametric version of Huelsenbeck and Bull's likelihood ratio test for conflicting phylogenetic signal [18]. In this test, a likelihood ratio test statistic is calculated for the ‘two tree’ versus ‘one tree’ models, and compared to a null distribution generated by non-parametric bootstrapping (see Methods). For our data, the test statistic, δ = 27.88, was markedly larger than any value from the null bootstrap distribution. This allows the null hypothesis to be firmly rejected with *p*<0.001 (figure 2B). Collectively these three tests show that the evolutionary histories of the host and HEG data significantly differ, and therefore provide evidence for the horizontal transfer of this HEG among these actinarians.

Having demonstrated that HEG horizontal transfer has very likely occurred, we were keen to estimate a rate of HEG movement: is this a frequent or rare event? Recent work based on fossil data suggests that an approximate age for the basal split of the host phylogeny in figure 1 is 500 MY [19]. Using branch lengths calculated by maximum likelihood, with a molecular clock enforced, the total time encompassed by the most likely resolution of the tree seen in figure 1 is approximately 5500 MY (estimated by summing the lengths of all the branches and converting to MY). We examined the dynamics of recurrent HEG gain and loss across this phylogeny using maximum likelihood methods as implemented by Pagel's Multistate (v0.8) program [20]. We found no significant difference between a one parameter model where rates of HEG gain and loss were constrained to be the same (ln likelihood = 13.478; 1 d.f.), and a two parameter model where rates were free to vary (12.691; 2 d.f.). A one parameter model which constrained gains to be zero was also significantly worse than a model which included gains (16.218, 1 d.f., *p*<0.025). The model where gains and losses were constrained to be equal produced an extremely tentative estimate of one transition (either gain or loss) every 500 MY, with 2 log likelihood support limits (equivalent to 95% confidence intervals) between once every 100 MY and 0 (never). Reconstructing specific transfer events between lineages is an extremely hard problem [21], but this method offers an alternative approach and provides a likely rate of movement. This very approximate estimate suggest that around 11 gain and loss cycles occurred across this phylogeny, and this equates to a very low horizontal transfer rate of 0.002 events per MY, with bounds from 0–0.01.

### HEG population study

One little studied area of HEG biology concerns their intra-population variance. All of the 95 *M. senile* individuals (from four UK collection sites, see Supporting Material 1) contained a complete HEG as indicated by the presence of an appropriately sized PCR product. All of the 51 different one-way sequences we obtained (approximately half and half 5′ and 3′) were identical to one another and identical to the published sequence from a single individual from the west coast of the USA [11].

## Discussion

The observation of functional, non-functional and absent HEG states, combined with our inference of horizontal transfer, strongly suggests that this HEG, like many others, conforms to a cyclical evolutionary model. These inferences suggest that this HEG invaded the Actinaria [6]. It is possible that whole mitochondrial genomes moved between species, but our supposition of HEG transfer is directly corroborated by a very recent study examining a number of complete anthozoan mitochondria: Medina et al [14] concluded this HEG (and not whole mitochondrial genomes) has been gained many times by the Hexacorallia (see figure 1). Our tentative estimate for a rate HEGs transfer among metazoans is approximately two orders of magnitude lower than that for HEGs in the mitochondria and nucleus of Saccharomycete yeasts [6], [7]. There are other reports of horizontal transfer events among metazoans, notably in insects because of the dynamics of other types of selfish element (e.g., TEs and P-elements in *Drosophila* [22], [23]). There are also reports of large mitochondrial horizontal transfer events and intron invasions of plants [8], [24]. Our conclusions are therefore not only consistent with other data concerning this HEG [14], but also with the idea that horizontal transfer and intron invasion occurs among multicellular organisms. These data suggest that, surprisingly, horizontal transfer rates on the order of once every 100 MY may be frequent enough to allow the invasion, cycling and persistence of HEGs within the Cnidaria.

Once transferred, the *COXI* HEG must home in order to restore purifying selection, and this requires that the mitochondria of the recipients must, at least on occasion, mix. The mitochondria of both mating types of the algae *Chlamydomonas* co-exist briefly after zygote formation before one is degraded, and this provides enough opportunity for an HEG to home [25] and therefore presumably invade a species where uni-parental mitochondrial inheritance seems the norm. Although we know of no direct evidence from sea anemones, mitochondrial leakage has been observed in a wide variety of animal species and may provide a window of opportunity for HEG homing [26].

Based on the distribution of this HEG (see figure 1), one has to infer that either the ancestor of the Cnidaria harboured an HEG, and that it has been lost a number of times, yet retained for some reason in the Hexacorallia, or else infer that the HEG was gained from some other lineage. Dalgaard et al [9] have reconstructed the relationships between over 130 HEGs, and the most closely related HEG to that in *M. senile* (apart from the sequences reported here) is found in *Neurospora crassa*. A seemingly strange best estimate would be that sea anemones received this HEG from fungi. However, this suggestion directly parallels data from angiosperms, which also appear to have gained a mitochondrial *COXI* HEG from fungi [8], [24] which then rapidly invaded the angiosperms via homing and horizontal transfer events.

The HEG survey results suggest that it is at or near fixation within *M. senile*. The lack of sequence divergence may imply a recent invasion and sweep within this species. However, it must be pointed out that the mitochondria of anthozoans are extraordinarily stable [27], with rates of evolution around only 0.03% per MY [28]. Moreover, the *COXI* genes of Anthozoans have extremely low levels of divergence [29], and one explanation for this highlights the discovery of a homologue to a mismatch repair gene [30].

Overall these data suggest that the HEG within the mitochondria of the Actinaria behaves in a similar evolutionary manner to HEGs in a variety of other organisms [1], [3], and conforms to the recurrent invasion model [6]. The low rates of horizontal transfer among metazoans will likely ensure that HEGs degenerate long before an opportunity to ‘escape’ arises, and mean that major clade-wide invasion is prevented. It might be that HEGs were able to invade anthozoan mitochondria only because of their extremely slow rates of substitution: this greatly extends the time to HEG degeneration, and damps down the periodicity of the HEG recurrent invasion cycle. It is only under these sluggish conditions that very rare horizontal transfer events are sufficient for long term HEG persistence and invasion. In general it seems that there need be a minimum lower bound on the rate of horizontal transfer, and frequency of homing opportunities, which enable HEGs to invade and persist in any clade. The likelihood of these events appears greater in simpler single celled organisms, but it also seems that whist the opportunities for homing and horizontal transfer are less in higher organisms, this does not necessarily put them out of HEGs reach. It is possible that HEGs were only able to invade sea anemones because of the slow evolutionary rate of their mitochondrial DNA [27], [28]. The extent to which HEGs have not invaded other metazoans may either be a lack of opportunity, or that most metazoans have faster substitution rates, which mean the more rapid degeneration of HEGs.

## Materials and Methods

### Sample collection

Twenty-nine different species of Cnidaria were sampled (see Table S1); these spanned the Anthozoa and Hydrozoa, but as a result of the tentative distribution of introns reported by Beagley et al [11] we mainly concentrated within the Anthozoa:Actinaria (true sea anemones). Samples were predominantly from the south coast of the UK but also included tropical examples. To investigate the within-species variation of HEGs we focused on *Metridium senile*. Ninety-five individuals from four different marinas on the south coast of the UK were sampled (see Supporting Material 1 for details).

### Molecular methods

When necessary DNA was isolated from tissues using the method described by Pinto et al [31]. HEG presence and absence was scored by amplification with the PCR primers described in Beagley et al [11]; these were designed such that the size of the product indicated the HEG status of each sample. The three 5′ most regions of the nuclear 18S and the ITS1-5.8S-ITS2 region were also amplified for each taxon using the primers described by White et al [32]. PCR products were directly sequenced and analysed with an ABI 3700 instrument. Complete and partial two-way sequences of the *COXI* intron were obtained for 14 of the intron containing taxa. Fifty-one one way *COXI* intron sequence were obtained from the 95 *Metridium senile* samples. Partial 18S sequence was obtained for all taxa but replaced with the more complete genbank 18S sequences AJ133552, AF254377, AJ877002, U19553, AY372249, AF052892 and AF052889 for *Actinia equina, Edwardsia gilbertensis, Lophelia pertusa, Tubularia indivisa, Sinularia sp, Palythoa variabilis* and *Metridium senile* respectively.

### Analyses

After manual inspection and editing where necessary, sequences were aligned using ClustalX [33]; the intron alignment was conducted by taking into account secondary structure following Goddard and Burt [6]. The resulting alignments were inspected and ambiguous areas were removed before phylogenetic analyses. The 18S data alone were insufficient to resolve some shallower nodes; we therefore added 5.8S and ITS data for these taxa since there was no conflict between data partitions. PAUP* (v 4.0b10) [34] and MrBayes (3.1.2) [35] for Windows were used for phylogenetic reconstruction. The appropriate likelihood models were selected using MrModeltest (v2.2) [36], and are shown in Supporting Material 1. Bayesian, likelihood and parsimony methods were used to analyse the host and HEG data sets. The loss test was conducted by constructing randomised intron assignments with Mathematica (v5.2) [37] and then using PAUP* to score the number of losses. Multistate (v0.8) [20] was used to estimate the most likely transition rates for HEG gain and loss across the host phylogeny.

### Phylogenetic congruence tests

To assess congruence between data partitions three methods were used. First, as a heuristic test, reciprocal Shimodaira-Hasegawa (SH) tests [15], as implemented in PAUP*, were used to test whether optimal trees from different partitions were incongruent. Congruence was more rigorously tested using a Bayesian model selection framework (Bayes factors) [16] and lastly, a modified version of the likelihood ratio test proposed by Huelsenbeck and Bull [18] (Leigh, Susko and Roger, unpublished) was employed.

The Bayes factor model selection was carried out with MrBayes as follows. Two models were tested. Under the ‘one tree’ model, the two data partitions were constrained to shared a single topology; however, the model parameters and branch-lengths were unlinked across the partitions for the Metropolis-coupled Markov chain Monte Carlo (MCMCMC) analysis. The ‘two tree’ model was identical to the ‘one tree’ model, except the tree topology was also unlinked, allowing tree-space to be separately explored for each data partition. The harmonic mean likelihood from the Metropolis-coupled Markov chain Monte Carlo (MCMCMC) iterations (after burnin was discarded) was used as an approximation to the marginal log-likelihood of the data [16]. For both the ‘one-tree’ and ‘two tree’ analyses, four independent runs from random starting trees were completed: two with one million MCMCMC iterations and two with two million MCMCMC iterations. Inspection of log-likelihood plots indicated that stationarity was achieved <50,000 iterations, and a conservative burnin of 150,000 iterations was discarded. Convergence was further assessed by monitoring the standard deviation in split frequencies between MCMCMC runs from different random starting trees. In all cases, at the end of the runs these standard errors were <0.01. Log-Bayes factors were computed by taking the differences in the harmonic mean log-likelihoods averaged over all four runs between the ‘two tree’ model and the ‘one tree’ model.

For the likelihood ratio test, the log-likelihood of two data sets (e.g. A and B) is maximized under the null hypothesis where A and B share a single tree (*T_AB_*) but each gene is allowed to have its own model and branch-length parameters (θ_A_ and θ_B_). The log-likelihood of this hypothesis is given by:
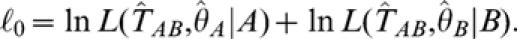
Under the alternative hypothesis each data set is allowed to have a separate tree (*T_A_* and *T_B_*) and its own model and branch-length parameters with maximum log-likelihood given by:
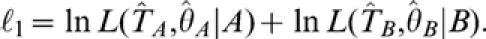
The log-likelihood ratio statistic (δ) is then defined as:
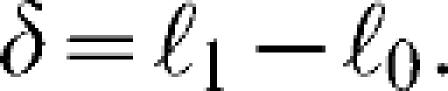
A null distribution for this statistic is generated by evaluating δ for 1000 non-parametric bootstrap resampled data sets, 50% of which are drawn entirely from data set A and 50% from dataset B. This procedure was accomplished with a software tool, CONCATERPILLAR (Leigh, Susko and Roger, unpublished) that calculates trees and likelihoods under the General Time Reversible plus invariable sites plus gamma model (GTR+I+Γ with the programs PHYML [38] and TREE-PUZZLE version 5.2 [39]).

## Supporting Information

Supporting Material 1 Details concerning the intra-specific sampling of *Metridium senile* and information concerning the models used in phylogenetic reconstruction, which were derived using Mr Model Test
(0.03 MB DOC)

Table S1 Table S1 shows the taxonomy, sampling location and personnel involved in the collection and identification of samples. The taxonomic hierarchy is mainly based on Fautin, Daphne G. 2005. Hexacorallians of the World. http://hercules.kgs.ku.edu/hexacoral/anemone2/index.cfm. ‘-’ indicates that this taxonomical level is not in common use in this taxa. Collection locations: ‘MBA tanks’ - sea water tanks of the Marine Biological Association of the UK, Plymouth; species may have recruited naturally from the circulating sea water system or been brought in from local shores by staff; ‘MBA standard haul’ is an offshore benthic trawl as described in Genner, M.J. et al Proc. R. Soc. Lond. B (2004) 271, 655-661; ‘Pet store’ - sample purchased from commercial pet store therefore original collection location is unknown. Personnel: ‘JB’ - John Bishop, MBA; ‘MD’ - Marymegan Daly, U. of Kansas, USA; ‘JD’ - Jo Davy, MBA; ‘SD’ - Simon Davy, MBA; ‘MD’ - Marie Le Goff, Southampton Oceanography Centre, UK; ‘KH’ - Keith Hiscock, MBA; and ‘KN’ - Ken Neal, MBA. All other collections and/or identification by Andrew Pemberton (AP) *Aglaophenia kirchenpaueri* - this species is known to occur at this location; however, the specimen was collected outside the reproductive season so lacked the gonads required for certain identification to species level.
(0.01 MB PDF)
