# Supplementary material for: Invasion and Persistence of a Selfish Gene in the Cnidaria
Source: PLoS One. 2006 Dec 20;1(1):e3. doi: 10.1371/journal.pone.0000003 (PMC1762336; doi:10.1371/journal.pone.0000003)
Supplement: Supporting Material 1 — Details concerning the intra-specific sampling of Metridium senile and information concerning the models used in phylogenetic reconstruction, which were derived using Mr Model Test (0.03 MB DOC) [file pone.0000003.s001.doc]

# Supporting material 1

***Intra-specific sampling of Metridium senile***

As *M. senile* is capable of clonal reproduction by pedal laceration we sampled floating marinas and collected one sample from each float. Colonisation of each float is by the sexually derived planktonic larval stage with adult movement between floats prevented by physical separation both from each other and the sea bed. Ninety-five individuals from four different marinas on the south coast of the UK were sampled: Plymouth (UK national grid reference SX459538), Brixham (SX929566) and two marinas on the river Hamble (SU485071 and SU485083). Minimum linear distance by sea between these sampled locations: Plymouth to Brixham, ~ 70 km; Brixham to Hamble, ~ 170km; Hamble to Hamble, ~ 1.3km.

***Models used in phylogenetic reconstruction derived from Mr Model Test***

**Cnidarian 18S, 5.8S and ITS model used to produce the 29 taxa tree seen in figure 1.**

Model selected: GTR+I+G

-lnL = 12086.9189

K = 10

AIC = 24193.8379

Base frequencies:

freqA = 0.2399

freqC = 0.2262

freqG = 0.2746

freqT = 0.2593

Substitution model:

Rate matrix

R(a) [A-C] = 0.8018

R(b) [A-G] = 2.3382

R(c) [A-T] = 0.8746

R(d) [C-G] = 1.3930

R(e) [C-T] = 2.8566

R(f) [G-T] = 1.0000

Among-site rate variation

Proportion of invariable sites (I) = 0.1182

Variable sites (G)

Gamma distribution shape parameter = 0.2735

**Cnidarian 18S, 5.8S and ITS model used to produce the 14 taxa tree seen in figure 2.**

Model selected: GTR+G

-lnL = 7604.5352

K = 9

AIC = 15227.0703

Base frequencies:

freqA = 0.2393

freqC = 0.2250

freqG = 0.2734

freqT = 0.2624

Substitution model:

Rate matrix

R(a) [A-C] = 0.7410

R(b) [A-G] = 2.4961

R(c) [A-T] = 0.9861

R(d) [C-G] = 1.7709

R(e) [C-T] = 2.2604

R(f) [G-T] = 1.0000

Among-site rate variation

Proportion of invariable sites = 0

Gamma distribution shape parameter = 0.1533

**HEG model**

Model selected: HKY+G

-lnL = 2650.4771

K = 5

AIC = 5310.9541

Base frequencies:

freqA = 0.3198

freqC = 0.1693

freqG = 0.2081

freqT = 0.3028

Substitution model:

Ti/tv ratio = 3.6316

Among-site rate variation

Proportion of invariable sites = 0

Gamma distribution shape parameter = 0.9004
