# Supplementary material for: Invasion and Persistence of a Selfish Gene in the Cnidaria
Source: PLoS One. 2006 Dec 20;1(1):e3. doi: 10.1371/journal.pone.0000003 (PMC1762336; doi:10.1371/journal.pone.0000003)
Supplement: Table S1 — shows the taxonomy, sampling location and personnel involved in the collection and identification of samples. The taxonomic hierarchy is mainly based on Fautin, Daphne G. 2005. Hexacorallians of the World. http://hercules.kgs.ku.edu/hexacoral/anemone2/index.cfm. ‘-’ indicates that this taxonomical level is not in common use in this taxa. Collection locations: ‘MBA tanks’ - sea water tanks of the Marine Biological Association of the UK, Plymouth; species may have recruited naturally from the circulating sea water system or been brought in from local shores by staff; ‘MBA standard haul’ is an offshore benthic trawl as described in Genner, M.J. et al Proc. R. Soc. Lond. B (2004) 271, 655-661; ‘Pet store’ - sample purchased from commercial pet store therefore original collection location is unknown. Personnel: ‘JB’ - John Bishop, MBA; ‘MD’ - Marymegan Daly, U. of Kansas, USA; ‘JD’ - Jo Davy, MBA; ‘SD’ - Simon Davy, MBA; ‘MD’ - Marie Le Goff, Southampton Oceanography Centre, UK; ‘KH’ - Keith Hiscock, MBA; and ‘KN’ - Ken Neal, MBA. All other collections and/or identification by Andrew Pemberton (AP) Aglaophenia kirchenpaueri - this species is known to occur at this location; however, the specimen was collected outside the reproductive season so lacked the gonads required for certain identification to species level. (0.01 MB PDF) [file pone.0000003.s002.pdf]

Class	Subclass	Order	Suborder	Tribe	Subtribe	Family	Genus and Species	Collection location	Sample provided by	Identification by
Anthozoa	Hexacorallia	Actinaria	Nynatheae	Thenaria	Acontiaria	Aiptasiidae	*Aiptasia mutabilis*	Tinside Pool, Plymouth Sound	SD	AP & KN
Anthozoa	Hexacorallia	Actinaria	Nynatheae	Thenaria	Endomyaria	Actiniidae	*Anemonia viridis*	Jennycliff Bay, near Plymouth		AP & KN
Anthozoa	Hexacorallia	Actinaria	Nynatheae	Thenaria	Endomyaria	Actiniidae	*Actinia fragacea*	Jennycliff Bay, near Plymouth		
Anthozoa	Hexacorallia	Actinaria	Nynatheae	Thenaria	Endomyaria	Actiniidae	*Actinia equina*	Renney Rocks, Plymouth Sound		
Anthozoa	Hexacorallia	Actinaria	Nynatheae	Thenaria	Endomyaria	Actiniidae	*Anthopleura balli*	MBA tanks		KN or SD
Anthozoa	Hexacorallia	Actinaria	Nynatheae	Thenaria	Endomyaria	Actinodendronidae	*Megalactis sp*	Taiwan	MD	MD
Anthozoa	Hexacorallia	Actinaria	Nynatheae	Thenaria	Endomyaria	Phymanthidae	*Phymanthus sp.*	Papua New Guinea	MD	MD
Anthozoa	Hexacorallia	Actinaria	Nynatheae	Thenaria	Acontiaria	Sagartiidae	*Cereus pedunculatus*	MBA tanks		AP & KN
Anthozoa	Hexacorallia	Actinaria	Nynatheae	Thenaria	Acontiaria	Sagartiidae	*Sagartia elegans*	MBA tanks		AP & KN
Anthozoa	Hexacorallia	Actinaria	Nynatheae	Thenaria	Acontiaria	Aiptasiidae	*Aiptasia sp.*	Pet store	JD	JD
Anthozoa	Hexacorallia	Actinaria	Nynatheae	Thenaria	Acontiaria	Metridiidae	*Metridium senile*	Genbank		
Anthozoa	Hexacorallia	Actinaria	Nynatheae	Thenaria	Acontiaria	Sagartiidae	*Actinothoe sphyrodeta*	Wembury Reef, near Plymouth		
Anthozoa	Hexacorallia	Actinaria	Nynatheae	Thenaria	Acontiaria	Hormathiidae	*Calliactis parasitica*	MBA standard haul		
Anthozoa	Hexacorallia	Actinaria	Nynatheae	Thenaria	Acontiaria	Hormathiidae	*Adamsia carcinopados.*	MBA standard haul		
Anthozoa	Hexacorallia	Actinaria	Nynatheae	Athenaria	-	Edwardsiidae	*Edwardsia gilbertensis*	Guam	MD	MD
Anthozoa	Hexacorallia	Actinaria	Nynatheae	Thenaria	Endomyaria	Thalassianthidae	*Heterodactyla sp.*	Papua New Guinea	MD	MD
Anthozoa	Hexacorallia	Corallimorpharia	-	-	-	Discosomatidae	*Amplexidiscus fensetrafer*	Pet store	MD	MD
Anthozoa	Hexacorallia	Corallimorpharia	-	-	-	Discosomatidae	*Discosoma sp.*	Pet store	JD	JD
Anthozoa	Hexacorallia	Scleractinia	-	-	-	Oculinidae	*Madrepora oculata*	Darwin sea mounts	MG	MG
Anthozoa	Hexacorallia	Scleractinia	-	-	-	Caryophylliidae	*Lophelia pertusa*	Darwin sea mounts	MG	MG
Anthozoa	Hexacorallia	Zoanthidea	-	-	-	Sphenopidae (Palythoidae)	*Palythoa* spp.	Pet store	JD	JD
Anthozoa	Octocorallia	Gorgonacea	-	-	-	Plexauridae	*Eunicella verrucosa*	Off Plymouth Sound	KH	
Anthozoa	Octocorallia	Alcyonacea	-	-	-	Alcyoniidae	*Sinularia spp.*	Pet store	JD	JD
Anthozoa	Octocorallia	Alcyonacea	-	-	-	Alcyoniidae	*Alcyonium digitatum*	MBA standard haul		
Hydrozoa	-	Hydroida	Athecata	-	-	Tubulariidae	*Tubularia indivisa*	Queen Anne's Battery marina, Plymouth	JB	
Hydrozoa	-	Hydroida	Thecata	-	-	Campanulariidae	*Laomedea angulata*	Off Drake's Island, Plymouth Sound	KH	KH & AP
Hydrozoa	-	Hydroida	Thecata	-	-	Plumulariidae	*Nemertesia ramosa*	MBA standard haul		
Hydrozoa	-	Hydroida	Thecata	-	-	Aglaopheniidae	*Aglaophenia kirchenpaueri*	Off Drake's Island, Plymouth Sound	KH	KH
Hydrozoa	-	Hydroida	Thecata	-	-	Sertulariidae	*Sertularia cupressina*	River Hamble, Hampshire, UK		
